# The Mediterranean Diet Positively Affects Resting Metabolic Rate and Salivary Microbiota in Human Subjects: A Comparison with the Vegan Regimen

**DOI:** 10.3390/biology10121292

**Published:** 2021-12-08

**Authors:** Simona Daniele, Giorgia Scarfò, Lorenzo Ceccarelli, Jonathan Fusi, Elisa Zappelli, Denise Biagini, Tommaso Lomonaco, Fabio Di Francesco, Ferdinando Franzoni, Claudia Martini

**Affiliations:** 1 Department of Pharmacy, University of Pisa, Via Bonanno 6, 56126 Pisa, Italy; simona.daniele@unipi.it (S.D.); lorenzo.ceccarelli@phd.unipi.it (L.C.); e.zappelli@studenti.unipi.it (E.Z.); claudia.martini@unipi.it (C.M.); 2 Department of Clinical and Experimental Medicine, University of Pisa, Via Savi 10, 56126 Pisa, Italy; g.scarfo1@studenti.unipi.it (G.S.); jonathan.fusi@med.unipi.it (J.F.); 3 Department of Biotechnology, Chemistry, and Pharmacy, University of Siena, 53100 Siena, Italy; 4 Department of Chemistry and Industrial Chemistry, University of Pisa, Via Moruzzi 13, 56126 Pisa, Italy; denise.biagini@dcci.unipi.it (D.B.); tommaso.lomonaco@unipi.it (T.L.); fabio.difrancesco@unipi.it (F.D.F.)

**Keywords:** Mediterranean diet, metabolic rate, oral microbiota, saliva, vegan diet

## Abstract

**Simple Summary:**

Salivary microbiota has been shown to be individualized and influenced by genetic and environmental factors, including macronutrient intake and lifestyle. Herein, the effect of two long-term dietary patterns, the Mediterranean and the vegan diet, was analyzed on oral microbiota composition and metabolic profile of human subjects. Moreover, we correlated microbial species to metabolic parameters. Subjects following the Mediterranean diet had a wider spectrum of oral bacteria and a better metabolic profile compared to the vegan diet, confirming the positive effects of a Mediterranean diet.

**Abstract:**

Salivary microbiota, comprising bacteria shed from oral surfaces, has been shown to be individualized, temporally stable, and influenced by macronutrient intake and lifestyle. Nevertheless, the effect of long-term dietary patterns on oral microbiota composition and the relationship between oral microbiota composition and metabolic rate remains to be examined. Herein, salivary microbiota composition and metabolic profile were analyzed in human subjects with vegan (VEG) or Mediterranean (MED) long-term dietary patterns. MED subjects presented significantly higher percentages of *Subflava* and *Prevotella* species as compared to VEG ones. Moreover, MED subjects showed a lower carbohydrate and a higher lipid consumption than VEG subjects, and, accordingly, a significantly higher basal metabolic rate (BMR) and a lower respiratory quotient (RQ). *Prevotella* abundance was demonstrated to be inversely related to RQ and carbohydrate consumption, whereas *Subflava* percentages were demonstrated to be positively correlated to BMR. *Lactobacillus* abundance, which was inversely related to *Subflava* presence in MED subjects, was associated with decreased BMR (Harris–Benedict) values. Overall, our data evidence the influence of macronutrient intake on metabolic profile and oral microbiota and confirm the positive effects of the Mediterranean diet on BMR and on the abundance of microbial species associated with a better macronutrient metabolism.

## 1. Introduction

The gut microbiota is a complex dynamic ecosystem composed by different microorganisms, including bacteria, fungi, viruses, and protists [1]. These microorganisms interact with each other and with the human host, and actively affect different host functions, including circadian rhythmicity, nutritional responses, metabolism, and immunity [2]. The microbiota composition is genetically determined, even though easily influenced by environmental factors, including behavioral habits (tobacco intake and drugs), hormonal fluctuation, diet, and physical activity [3]. Among these, diet has a remarkable role in regulating and balancing the microbiota composition; for example, a low-fiber high-fat/high-carbohydrate diet (typical of Western countries) is often associated with intestinal dysbiosis, as opposed to the Mediterranean diet, with its high fiber content and richness in antioxidant molecules that reduce the growth of some pathogens such as *E. coli* and other Enterobacteriaceae [4]. Similarly, the vegan diet causes higher remarkable changes to the gut microbiota in terms of composition compared to an omnivore regimen. Despite the risk of an insufficient caloric intake, the vegan diet is usually rich in fibers, polyphenols, and antioxidant vitamins, and these nutrients positively affect the intestinal microenvironment [5]. In fact, the notable amount of non-digestible fibers seem to favor the growth of lactic acid bacteria (*Ruminococcus*, *E. rectale*, and *Roseburia*), while *Clostridium* and *Enterococcus* species are reduced [6]. Moreover, the increased polyphenol intake favors the growth of *Bifidobacterium* and *Lactobacillus* with consequent anti-inflammatory effects [6].

Although the impact of a vegan diet on the intestinal microbiota is well-documented, little is known about its influence on the oral microenvironment. The salivary microbiota refers to non-pathogenic microorganisms that are detected in saliva and cover the oral cavity surface, especially the tongue [7]. The importance of analyzing oral communities lies in their ability to interact with the whole-body commensal microflora, positively affecting human health. In particular, the salivary dysbiosis has been shown to be related to some oral diseases (such as caries, periodontitis, mucosal alterations, and oral cancers) [7]. This bacterial population has been demonstrated to be individualized; saliva composition, due to its high content of antimicrobial substances, plays a crucial role in shaping resident populations [8]. As far as the impact of diet is concerned, it has already been demonstrated that the oral bacterial population of infants is strictly related to their breast-milk diet and that changes in microbiota composition depend on diet variations [9]. Although these data are significant, the milk diet is age-related and substantially time-limited. In fact, little is known about the effects of prolonged diet habits on oral microbiota and consequent human health. Some nutrients have been shown to influence the oral microbial pattern. Among them, high fiber and fatty acid intake positively influence the diversity and community structure of the salivary microbiota [10]. It has been reported that salivary metabolomes are discriminant of omnivore, ovo–lacto–vegetarian and vegan individuals but the difference in their oral microbiota composition is unclear [11].

Besides altering human microbiotas, diet quality or macronutrient intake adequacy may affect body composition and weight changes [12]. In particular, the intake of simple sugars and some saturated fatty acids has adverse effects on body adiposity. In contrast, protein and fiber consumption seem to beneficially modulate satiety and energy metabolism-related processes [13]. Nevertheless, a clear relationship between diet regimen and human metabolic profile has not yet been elucidated. In this sense, genetic background and gut microbiota composition can be contributing factors to metabolic inter-individual differences in macronutrient consumption [13].

In the present study, we aim to elucidate the impact of two different long-term dietary patterns, vegan and Mediterranean diets, on the salivary microbiota composition. In addition, the same diets were evaluated for their influence on the metabolic rate. Advances in understanding of the composition of the microbiota and the metabolic pathways related to macronutrients involved in energy production, may be of great benefit to precision nutrition and public health.

## 2. Materials and Methods

Forty-two subjects (20 males and 22 females; age 38 ± 1) were enrolled in the study. The population was divided according to eating habits, Mediterranean diet or vegan diet, using a validated food habits questionnaire (FHQ) [14]. These dietary patterns had to be followed for at least 2 years. Subjects were recruited during sport physical exams performed at the Department of Clinical and Experimental Medicine of the University of Pisa. This study was carried out in accordance with the recommendations of Declaration of Helsinki and Great North-West Area of Tuscany guidelines, with written informed consent from all subjects. The protocol was approved by the Great North-West Area of Tuscany (152/2016).

The FHQ indicated the frequency of nutrient consumption and each food was categorized as follows: never or less than once per month, one to three times per month, once per week, one to four times per week, five to six times per week, once per day, two to three times per day, four to five times per day, or six times or more per day [14]. Of note, most of the enrolled vegan subjects did not agree to answer all the questions related to their nutritional regimen. These subjects give information on the type of carbohydrate source, fatty acids, and vegetables, and finally the protein source, in the specific case of legumes, without mentioning the quantities in grams. The lack of information on the grams’ intake of macro-nutrients did not allow the calculation of both caloric and protein intakes.

Following a comprehensive medical history, subjects underwent spontaneous saliva sampling (3 mL) and basal metabolic assessment by indirect calorimetry. Basal metabolic rate (BMR) was used to outline the metabolic profile of the enrolled population. BMR indicates the minimum energy required to maintain all the vital activities and functions in the awake state [15]. This is estimated through several equations; among them, the Harris–Benedict equation is a good method to predict BMR taking into account anthropometric factors, including age, weight, and height [16].

### 2.1. Indirect Calorimetry

Indirect calorimetry is a good method for assessing energy expenditure at rest (basal metabolic rate (BMR) or respiratory exchange ratio (RER)) and identifying the percentages of energy substrates used—carbohydrates (CHO) and free fatty acids (FFA) [17]. The substrate used for energy is useful to understand the metabolic profile and the possible relationship with risk factors such as diabetes and insulin resistance [18]. The subjects were in controlled environmental conditions (temperature of 22–24 °C) 48 h after the last workout. A paramagnetic or fuel-cell O_2_ sensor and an infrared CO_2_ analyzer were purchased from Quark FPT (COSMED Srl, Albano Laziale, Rome, Italy). The subjects were laid out on a couch, after which a canopy helmet (CANOPY) was positioned for gas analysis. After an acclimatization period of about 5 min, the evaluation began and lasted 20 min. The acclimatization period was essential to ensure that no hyperventilation occurred which could influence our data. The gas analysis was measured by a breath-by-breath method. The test was conducted under medical supervision.

### 2.2. Bacterial DNA Extraction and RT-PCR

Total bacterial DNA was extracted from 3 mL of saliva samples using QIAamp BiOstic Bacteremia Kit (QIAGEN, Milan, Italy) according to the manufacturer’s protocol. Extracted DNA was quantified using NanoDrop (Thermofisher, Milan, Italy) and for each sample, 1 and 2.5 µg of DNA was amplified with MiniOpticon (BIORAD, Milan, Italy). RT-PCR reactions consisted of 10 μL Fluocycle^®^ II SYBR^®^ (Euroclone, Milan, Italy), 0.6 μL of both 10 μM forward and reverse primers, 5 μL cDNA, and 3.8 μL of H_2_O. All reactions were performed for 40 cycles using the following temperature profiles: 98 °C for 30 s (initial denaturation); T °C (see Table 1) for 30 s (annealing); and 72 °C for 3 s (extension). PCR specificity was determined by both the melting curve analysis and gel electrophoresis.

### 2.3. Quantification of Absolute and Relative Bacterial DNA

The content of bacterial DNA derived from specific microorganisms, among which *Streptococcus Pneumoniae, Neisseria Subflava*, all *Lactobacillus* genera, and *Prevotella,* were analyzed. The primers used are summarized in Table 1. The absolute quantity of total bacterial DNA was determined by qPCR using a calibration curve, *CT* = f(log_10_[pgDNA]), generated with a known concentration of a standard bacterial DNA and primers for the conserved region of the bacterial 16S rRNA gene (Table 1). The calculated quantity of bacterial DNA was used to determine a ‘corrective factor (CF)’ for each saliva sample. This CF has allowed us to calculate the exact quantity of bacterial DNA with respect to the quantity of DNA measured with NanoDrop, which was revealed to contain a not negligible amount of human host DNA. The CF indicates the fraction of bacterial DNA relative to the total DNA extracted with the kit.

The absolute amount of bacterial DNA derived from each microorganism was also determined by qPCR using a calibration curve generated for each specific primer pair. In these cases, the real amount of bacterial DNA loaded in the qPCR mix was calculated by multiplying the total DNA loaded by the CF and the result was used to calculate the relative amount of DNA derived from each microorganism to the total bacterial DNA.

### 2.4. Statistical Analysis

The GraphPad Prism (GraphPad Software Inc., San Diego, CA, USA) was used for graphical presentations. All data are presented as the mean ± SEM. Statistical analyses were performed by Mann–Whitney unpaired *t*-test. *p* < 0.05 was considered as statistically significant. Covariate analysis was performed by the z test. All statistical procedures were performed using the StatView program (Abacus Concepts, Inc., SAS Institute, Cary, NC, USA).

## 3. Results

### 3.1. Descriptive Statistics

The clinical characteristics of the enrolled subjects (subjects with vegan diet, VEG, and subjects with Mediterranean diet, MED) are reported in Table 2. We obtained the data by analyzing the FHQ using the score obtained from the food frequency of food ingestion.

The VEG and MED cohort were sex-matched and presented a mean age of 33.6 ± 12.4 and 31.9 ± 9.0, respectively. The two groups did not present significant differences in body mass index (BMI, *p* = 0.2246).

### 3.2. Basal Metabolic Rate Evaluation

BMR was significantly higher in subjects following a MED diet than in those with a VEG one (*p* = 0.0008).

The measured respiratory quotient (RQ) was significantly higher in subjects with a VEG diet than in those with MED one (*p* = 0.0189).

Furthermore, the percentage of total consumption of carbohydrates (CHO%) was significantly lower in the presence of a MED diet with respect to a VEG regimen (*p* = 0.0093). Consistent with these data, the total consumption of lipids (Lipids%) was significantly higher in subjects with a MED diet than a VEG one (*p* = 0.0134).

As expected, BMI positively correlated with CHO% (*p* < 0.0001) and negatively related to Lipids% (*p* < 0.0001) in the MED subgroup. Of note, these correlations did not reach significance in the VEG subgroup (see Discussion section).

Of note, most of the enrolled vegan subjects did not agree to give information on the quantities in grams of the different macronutrients. The lack of information on the intake of macro-nutrients did not allow for the calculation of both caloric and protein intakes.

### 3.3. Oral Microbiota Analysis

**Figure 1:**
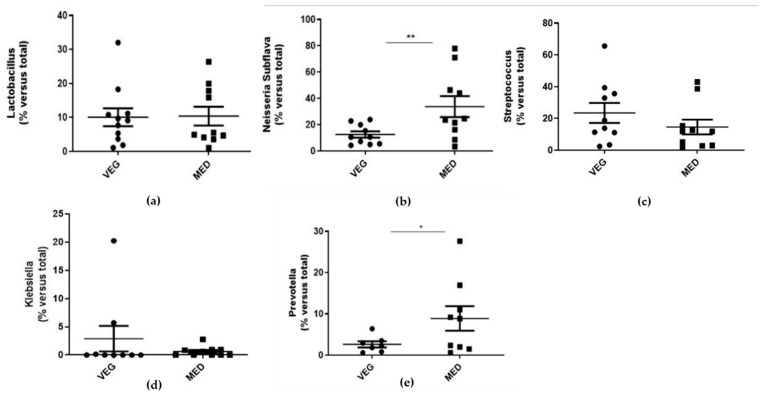
(**a**–**e**): Evaluation of salivary bacterial expression. The salivary samples were used for the extraction of bacterial DNA using the appropriate commercial extraction kit (QIAamp BiOstic Bacteremia Kit, Qiagen). The levels of each individual bacterium were detected with the use of specific primers towards Lactobacillus (**a**), Subflava (**b**), Streptococcus (**c**), Klebsiella (**d**), and Prevotella (**e**), by real-time PCR analysis. In parallel, the 16S primer was used to obtain the total percentage of bacterial DNA, in order to determine a ‘correction factor (CF)’ for each saliva sample. Data are reported as the mean values ± SD; statistical analysis was performed by unpaired *t*-test: * *p* < 0.05, ** *p* < 0.01 versus total population.

A real-time PCR analysis was performed to determine the relative abundance of salivary microorganisms (Figure 1; Table 3).

The presence of *Lactobacillus* (*p*= 0.9018), *Streptococcus* (*p* = 0.1077), and *Klebsiella* (*p* = 0.1496) was found to be comparable between the two groups. The abundance of *Subflava* (*p* = 0.007) and *Prevotella* (*p* = 0.0127) was significantly higher in MED subjects compared to VEG ones.

All the microbial species were correlated to each other by linear regression analysis (Table 3). In the enrolled population, Lactobacillus was positively correlated with Streptococcus (*p* = 0.0001) and inversely related to *Subflava* (*p* = 0.0144). The latter correlation was lost in VEG subjects (*p* = 0.4321), who, in contrast, presented a negative correlation between *Streptococcus* and *Prevotella* specimens (*p* = 0.0010).

No other significant correlation was found between the microorganisms analyzed in the present study.

### 3.4. Correlation between Metabolic and Microbiota Parameters

All the microbial species were correlated with the metabolic parameters by linear regression analysis (Table 4).

No significant associations were found between *Prevotella* and BMI in the total population. Interestingly, *Prevotella* was positively related to BMI in subjects following a VEG diet (*p* = 0.0004) and inversely correlated in those who followed a MED diet (*p* = 0.0195). Consistent with the association of the latter, *Prevotella* abundance was inversely related to RQ (*p* = 0.0429) and CHO% (*p* = 0.0430) and positively related to lipid consumption (*p* = 0.0481).

The relative abundance of *Subflava* specimens was positively related to BMR Harris–Benedict in the total population of human subjects (*p* < 0.0001).

In contrast, no significant correlation was found in the whole population between Lactobacillus abundance in saliva and BMR Harris–Benedict (*p* = 0.4529). Interestingly, we found that Lactobacillus specimens were positively correlated with BMR Harris–Benedict in VEG subjects (*p* = 0.0137) and inversely correlated in MED ones (*p* = 0.0238), further evidencing the relationship between salivary microbiota and metabolic rate (in this respect, see Discussion section).

## 4. Discussion

Herein, the metabolic profile and the salivary microbiota composition were analyzed in human subjects. The present study aimed to dissect the impact on the salivary microbiota and on the metabolic profile of two different long-term dietary patterns, i.e., vegan and omnivore diets. The main findings of our work are as follows: (i) the Mediterranean diet was associated with a significantly higher BMR and a lower RQ, and with a lower carbohydrate consumption and a higher lipid consumption with respect to the vegan diet; (ii) MED subjects presented significantly higher percentages of Subflava and Prevotella species compared to VEG subjects; and (iii) salivary Lactobacillus abundance was positively correlated with *Streptococcus* and inversely related to Subflava abundance.

In addition, when microbial species were correlated with metabolic parameters, we found that: (i) Subflava specimens were positively related to BMR Harris–Benedict; (ii) Prevotella abundance was inversely related to BMI (in MED subgroup), to RQ, and to CHO%, and positively related to Lipids%; and (iii) Lactobacillus abundance was positively correlated with BMR Harris–Benedict in VEG subjects and inversely correlated in MED ones.

Overall, our data evidences the influence of macronutrient intake on metabolic profile and oral microbiota and confirms the positive effects of the Mediterranean diet on basal metabolic rate and on the abundance of microbial species associated with a better macronutrient metabolism.

Macronutrient intake can consistently shift the composition of the gut microbiota, which has been shown to be primarily influenced by fibers contained in fruits, vegetables, and other plant foods [6]. The salivary microbial profile can be considerably influenced by nutrient intake, life-style, and even circadian rhythm, as well as with changes in body weight, cortisol rhythm, basal metabolic rate, glucose tolerance, and body temperature [6]. Nevertheless, the majority of studies have been conducted in animals, and further investigations are needed to elucidate the link between different dietary regimens and salivary microbiota. Moreover, a further objective of the study was to unveil the putative relationship between changes in microbial composition and metabolic rate.

The main features of the vegan diet are a decreased fat intake and high complex carbohydrate consumption. Although these factors seem to be protective against chronic diseases, little is known about the impact of a vegan dietary pattern on BMR [19].

In our population, subjects following the Mediterranean diet had a significantly higher BMR compared to subjects with the vegan diet, as demonstrated previously in animals—Carnivora usually have an elevated BMR compared to vegetarian species [20]. The different metabolic rates between VEG and MED subjects can be explained by considering the macronutrient intake, and in particular, the lower protein consumption in those who followed a VEG diet [20]. Moreover, VEG subjects have been demonstrated to ingest a lower amount of iodine, with a consequently reduced production of thyroid hormones (T3 and T4) [21], that are notably associated with an efficient BMR [22].

Consistent with a lower BMR, VEG subjects presented a significantly higher RQ compared to MED ones, indicating lower fat oxidation and higher carbohydrate oxidation [23]. This result reflected the percentage of total consumption of carbohydrates that was significantly higher in VEG individuals. Although a low fat oxidation has been associated with weight gain and obesity [24], the two groups did not present significant differences in BMI. This result may be explained considering that fat storage and weight gain are not influenced exclusively by RQ, but mostly depend on the energy balance [25].

As expected, BMI was positively correlated with CHO% and negatively related to lipids % in MED subgroup. Of note, these correlations did not reach significance in the VEG subgroup, thus suggesting that different metabolic adaptations can occur in this diet regimen, for which further investigations are surely needed. In this sense, the body composition and insulin sensitivity may help in explaining this discrepancy.

Of note, the estimation of the caloric intake, as well of the macro- and micro-nutrient amounts, would add important information on the effect of the two diet regimens on metabolic rate. Unfortunately, most of the enrolled vegan subjects did not agree to give information on the quantities in grams of the introduced macronutrients.

In light of these considerations, the data concerning BMR and RQ can, however, be considered as a useful tool to analyze the population, underlining the differences between vegan and Mediterranean diets. Of note, we completely agree with the reviewer that the collection of data on caloric or protein intakes would add significant knowledge on the type of effect of the two diet regimens.

When the salivary microbiota was analyzed, the data showed that MED subjects presented significantly higher percentages of Subflava and Prevotella species compared to VEG ones. Several factors could influence differences in the oral microenvironment. Among them, different bacterial intakes and their substrate consumption, seem to affect microbiota composition [6]. In addition, plant-based nutrition provides a remarkable amount of medium-chain fatty acids, unsaturated fatty acids, and fibers that influence microbial species growth. In contrast to our study, the vegan diet has been shown to favor Subflava and Prevotella species [10] when compared to omnivorous diets. Actually, it should also be considered that the Mediterranean diet is rich in fruits and vegetables with a high content of fibers [26]. This feature may explain the abundance of Subflava and Prevotella found in our subjects, compared to VEG. Consistent with our results, a previous study had already demonstrated that the Mediterranean diet, with its high intake of short-chain fatty acids, favored the abundance of Prevotella in the gut microbiota [27].

In addition, a plant-based diet provides a greater intake of inorganic nitrate with a consequent growth of bacteria that are able to reduce nitrate to nitrite. Among these, Prevotella, a great contributor to nitric reduction, seems to be particularly abundant in VEG subjects compared to omnivorous ones [28]. In our population, the remarkable vegetable intake in those who followed the Mediterranean diet may be at the basis of a high nitrate bioavailability that favors Prevotella growth. Consistent with our result, a previous study suggested that the vegan diet may not influence nitrate and nitrite homeostasis since no significant differences have been found in terms of plasma and salivary concentrations between VEG and omnivorous subjects [28].

Microbial species were also correlated with metabolic parameters. In our population, Subflava was positively related to BMR Harris–Benedict reflecting the variety of the Mediterranean diet. Considering that many species of Neisseria, including Subflava, are unable to utilize many carbohydrates [29], microbial species in the general population (VEG + MED subjects) seem to use a metabolism based mostly on lipid consumption which guarantees a higher BMR [30]. In the total population, Prevotella was found to be inversely related to CHO consumption, in line with the fact that it is a polysaccharide-degrading bacteria [30]. Moreover, the vegan diet provides a large amount of polysaccharides, that favor Prevotella abundance [30] but increase energy intake contributing to gaining weight [31]. In murine models, Prevotella abundance in gut microbiota, was associated with insulin resistance and with a worse metabolic profile, thus explaining the positive correlation between Prevotella and BMI that was evidenced in VEG subjects [32]. This correlation has been already highlighted in obese children in which a decrease in the gut Bacteroides/Prevotella ratio (meaning a relative abundance of Prevotella) was associated with a higher BMI [33].

Interestingly, abundance of Prevotella was inversely correlated with BMI in MED subjects. A similar result has already been shown in a previous study focused on gut microbiota composition; in particular, a relative abundance of Prevotella in the intestinal microenvironment has been associated with better anthropometric features, among these a lower BMI [34].

In examining the composition of the oral microbiota, most studies have focused on Lactobacillus samples [35] because of their importance in carbohydrate and lipid metabolism [36]. Herein, Lactobacillus abundance was related to metabolic rate. In particular, BMR was positively related to BMR in VEG subjects. In this sense, the vegan diet has been shown to promote Lactobacillus abundance and weight loss in the gut microbiota [6,36], possibly favoring an increase in BMR.

In contrast, Lactobacillus specimens were inversely related to BMR in the MED subgroup. This paradox may be explained considering that the two regimen diets may favor the growth of different species of *Lactobacillus*, by introducing animal- or plant-derived foods. In this sense, selected *Lactobacillus* species have been demonstrated to be implicated differently in the glycogen metabolic pathway [37] and thus in the host metabolic rate.

## 5. Conclusions

Overall, our data suggest that a long-term dietary pattern could affect both metabolism and salivary biodiversity. In this case, the Mediterranean diet with a higher content of proteins and lipids, is associated with a better metabolic profile when compared to the vegan one. In addition, nutrient variety in the Mediterranean diet is mirrored in a wider oral microbial population, itself correlated with a beneficial metabolic panel. Future work will examine the peculiar correlation between physical-exercise-related RQ and microbiota composition.

## Figures and Tables

**Table 1:** Nucleotide sequences and annealing temperature of the primers utilized in PCR experiments.

	Reverse	Forward	T Annealing
*16S*	AGGGTTGCGCTCGTTG	GTGCCAGCAGCCGCGGTAA	64 °C
*All Lactobacillus*	CCACCTTCCTCCGGTTTGTCA	AGGGTGAAGTCGTAACAAGTAGCC	60 °C
*Nesseria subflava*	TGGAAGACGGATTTGGTGTAAT	CCAACGATGTTGCCGAATTG	58 °C
*Streptococcus pneumoniae*	GTACAGTTGCTTCAGGACGTATC	ACGTTCGATTTCATCACGTTG	55 °C
*Prevotella*	GTGGCGCGTATTTTATGTATGTG	ATCCGCCATACGCCCTTAG	60 °C

**Table 2:** Descriptive statistics of clinical parameters for VEG and MED subjects. The data are expressed as mean ± SD. Statistical analysis was performed by unpaired *t*-test. * *p* < 0.05, ** *p* < 0.01, vs. VEG subjects. BMR: basal metabolic rate; RQ: respiratory quotient; CHO%: carbohydrate consumption; Lipids%: lipid consumption.

Group	Age	Weight	Height	BMI	BMR Harris– Benedict	BMR	RQ	CHO%	Lipids%
VEG (Mean ± SD)	33.6 ± 12.3	60.6 ± 12.3	165.3 ± 7.2	22.1 ± 4.2	1425.5 ± 134.8	1233.6 ± 417.8	0.8 ± 123	48.08 ± 33.2	53.03 ± 33.9
MED (Mean ± SD)	31.7 ± 8.9	72.6 ± 16.9	174.1 ± 8.6	23.6 ± 3.5	1677.8 ± 295.7 **	1630.5 ± 566.9 *	0.7 ± 0.037 *	25.6 ± 16.3 **	74.7 ± 16.5 *

**Table 3:** Descriptive statistics of microbial parameters for VEG and MED subjects. The data are expressed as mean ± SD. Statistical analysis was performed by unpaired *t*-test. * *p* < 0.05, ** *p* < 0.001 vs. VEG subjects.

Group	*Lactobacillus* (%/Total)	*Subflava* (%/Total)	*Streptococcus* (%/Total)	*Klebsiella* (%/Total)	*Prevotella* (%/Total)
VEG (Mean ± SD)	10.08 ± 8.5	12.6 ± 7.3	23.4 ± 19.2	2.7 ± 6.4	2.6 ± 1.8
MED (Mean ± SD)	10.4 ± 8.5	33.7 ± 24.7 **	14.6 ± 14.2	0.6 ± 0.8	8.8 ± 8.6 *

**Table 4:** Correlation between microbial species and metabolic parameters in the total population, VEG subjects, and MED subjects; correlation between parameters was determined by simple linear regression analysis, using the StatView program (Abacus Concepts, Inc., SAS Institute, Cary, NC, USA). Z and *p* values obtained for each correlation are reported in the respective column.

	Total Population			VEG			MED		
	Correlation	Z-Value	*p*-Value	Correlation	Z-Value	*p*-Value	Correlation	Z-Value	*p*-Value
*Lactobacillus, Streptococcus*	0.559	3.838	0.0001	0.803	4.563	<0.0001	0.336	1.440	0.1500
*Lactobacillus, Subflava*	−0.382	−2.448	0.0144	−0.188	−0.786	0.4321	−0.663	−3.294	0.0010
*Lactobacillus*, BMR Harris–Benedict	−0.120	−0.751	0.4529	0.512	2.464	0.0137	−0.499	−2.261	0.0238
Subflava, BMR Harris–Benedict	0.647	4.679	<0.0001	0.159	0.659	0.5096	0.592	2.808	0.0050
*Klebsiella*, BMI	−0.375	−2.362	0.0182	−0.406	−1.725	0.0846	−0.433	−1.909	0.0563
*Klebsiella*, CHO%	0.312	1.939	0.0525	0.287	1.179	0.2382	−0.207	−0.866	0.3863
*Klebsiella*, Lipids%	−0.315	−1.954	0.0507	−0.291	−1.198	0.2309	0.194	0.808	0.4190
*Prevotella*, CHO%	−0.359	−2.024	0.0430	−0.609	−2.347	0.0189	−0.335	−1.348	0.1777
*Prevotella*, BMI	−0.099	−0.537	0.5913	0.792	3.574	0.0004	−0.539	−2.336	0.0195

## Data Availability

The row data presented in this study are available on request from the corresponding author.
